# The Causes and Treatment of Diarrhœa—II

**Published:** 1908-11-07

**Authors:** 


### Medicine.

### THE CAUSES AND TREATMENT OF DIARRHŒA—II.

Although cases of ptomaine poisoning can be divided up in the way already described into two groups—those without, and those with, a latent period, and although these correspond fairly accurately with cases in which ptomaines are eaten ready formed, and cases in which the patient ingests no actual ptomaines but bacteria ready to produce ptomaines: nevertheless the two groups may overlap. For instance, a man might eat food containing both ptomaines and ptomaine bacteria; he would then develop immediate symptoms due to the former, might seem to be recovering satisfactorily, and yet have a return of all his symptoms with increased severity somewhere between eight and twenty-four hours later. The type of the attack or attacks may show the very greatest variability in this respect, according to the different proportions of ready-formed ptomaines, and of ptomaine-producing bacteria ingested respectively.

In the next place, it is clearly easier to eradicate a finite quantity of a poison, than it is to get rid of organisms which may go on multiplying and producing ptomaines indefinitely. Hence the prognosis is very much better in the cases in which ptomaines are ingested without microbes, than it is in the cases in which microbes without ready-formed ptomaines are taken in.

Lastly, although more than one kind of micro-organism may produce ptomaines, one of the commoner varieties to do so in man is the *Bacillus enteritidis* of Gaertner. The importance of this arises from the fact that the serum of a patient infected by Gaertner's bacillus develops the power of agglutinating cultures of that organism. In cases of doubt, therefore, the reaction of the patient's serum to the bacillus enteritidis may be tested in a manner precisely similar to Widal's test for typhoid fever; if the reaction is positive the diagnosis is confirmed in a way that may subsequently be of the greatest importance medico-legally. The reaction may be negative without excluding infection by Gaertner's bacillus, but a positive reaction is good proof of infection by this micro-organism. Like most agglutinating reactions it rapidly passes off again as the patient recovers his normal health.

(2). *Diarrhœa due to abnormal conditions of the bowel walls.*—Inflammation, ulceration, and amyloid degeneration of the walls of the intestine (large or small) often have diarrhœa as their most characteristic symptom. The diarrhœa may not be constant, but it is very readily produced under these circumstances by food substances which in a normal intestine cause no irritation at all.

Amyloid degeneration may be dismissed in a few words, for it is decidedly uncommon nowadays. Chronic phthisis and tertiary syphilis are now the only causes of lardaceous disease that are at all common. Diarrhœa in cases of chronic phthisis would be quite as likely to be due to tuberculous ulceration of the intestine as to lardaceous disease, even supposing albuminuria and enlargement of both liver and spleen helped to indicate the latter. In tertiary syphilis, on the other hand, severe and persistent diarrhœa in the absence of rectal ulceration would be a point in favour of amyloid degeneration, if there was corresponding evidence of it in kidneys, liver, and spleen.

Inflammation of the intestine—enteritis and colitis—may be due to many different causes, and may assume different forms. It will suffice to indicate a few of these. There is the variety due to metallic irritants, such as arsenic, calomel, or corrosive sublimate, by which an intense irritation may be produced, passing on to acute inflammation and even ulceration. Indigestible and decomposing food substances may do the same sometimes. Then there are the inflammations and ulcerations of the bowel produced by special organisms—cholera, amœbic dysentery, bacillary dysentery, sprue in the tropics, ulcerative colitis or English dysentery at home; all of these may begin with severe diarrhœa as their first symptom, and most of them may become chronic, in which case persistent diarrhœa is still the most prominent and troublesome result. Chronic dysentery and chronic ulcerative colitis are sometimes described as separate diseases, but to all intents and purposes they are the same, the former arising in tropical countries as an acute illness which does not entirely resolve, the latter developing at home in patients who have never been abroad. Mucous colitis or mucous colic is an entirely different condition which is almost unmistakable, and although diarrhœa is sometimes complained of constipation is far more marked a feature; the diarrhœa only comes on occasionally, and after adequate causes such as the taking of purgative drugs.

It remains to mention the other ulcers of the bowel that may cause diarrhœa—tuberculous, typhoidal, venereal, malignant. There are certain main points that require notice in regard to each of these. In tuberculous ulceration of the bowel diarrhœa is quite exceptional; if it does occur, however, it is often severe and very difficult to check. But in the majority of cases there is no symptom whatever during life to indicate this lesion; there may be thirty or forty large tuberculous ulcers present without any diarrhœa. It is none the less possible to arrive at the diagnosis with fair accuracy however, for post-mortem findings prove that cases of phthisis in which there is tuberculosis of the larynx invariably have ulcers in the intestine also.

In the next place diarrhœa in typhoid fever is decidedly the exception rather than the rule. More typhoid cases suffer from constipation requiring evacuant enemata than are afflicted by diarrhœa requiring starch and opium enemata. Diarrhœa in typhoid fever usually occurs either quite early in the illness before the diagnosis is clear, castor oil having been given to relieve temporary constipation; or else, later in the illness, when the digestive powers are so impaired that ordinary milk begins to pass through the bowel undigested. It is always important to look for curds in the stools in cases of typhoidal diarrhœa.

